# The Impact of COVID-19 on Menstrual Cycle in Women

**DOI:** 10.3390/jcm12154991

**Published:** 2023-07-29

**Authors:** Grzegorz Błażejewski, Joanna Witkoś

**Affiliations:** Faculty of Medicine and Health Science, Andrzej Frycz Modrzewski Krakow University, G. Herlinga-Grudzińskiego, Street 1, 30-705 Kraków, Poland

**Keywords:** menstrual disorders, COVID-19, long COVID, COVID-19 vaccine, women

## Abstract

Background: The COVID-19 pandemic has become the largest and most diverse to threaten the health of humanity since the 1918 influenza pandemic. Methods: This study involved 113 women who had suffered from COVID-19. The study was conducted as interviews with each woman during visits to a clinic prior to the start of their post-COVID-19 physiotherapy treatment cycle. The aim of this study was to assess the prevalence of changes in the women’s monthly cycles related to COVID-19, as well as to analyse correlations between dependent variables relating to changes in the monthly cycle and independent variables relating to other factors, such as age, weight, number and type of vaccinations, and time since illness. Additionally, the study assesses correlations between the monthly cycle and COVID-19 symptoms persisting after the illness (long COVID). Results: Women who reported more symptoms of COVID-19 were more likely to report changes in their menstrual cycle occurring after the SARS-CoV-2 infection, compared with women whose disease course was mild. Women who declared that COVID-19 affected their monthly cycles most often indicated increases in abdominal, lower abdominal, and joint and muscle pain, as well as in the severity of headaches during monthly bleeding. A small percentage of women indicated that their monthly cycles were longer and their regularity disrupted. Conclusions: This study shows that the more COVID-19 symptoms a woman had, the more often there were noted changes in monthly cycle. The same relationship was also found for persistent long COVID symptoms. The longer the time lapse since the COVID-19 infection, the less frequently changes in the monthly cycle were recorded.

## 1. Introduction

Almost three years have passed since 11 March 2020, when the World Health Organization (WHO) declared the global pandemic of COVID-19, caused by a member of the RNA coronavirus family SARS-CoV-2 [1,2]. Despite huge efforts on the part of all countries to undertake vaccination programmes in the fight against SARS-CoV-2, unfortunately, the pandemic is still ongoing and new COVID-19-related deaths are reported every day [3,4]. The weekly epidemiological update on COVID-19 issued by the WHO stated that as of 2 July 2023 there have been 767 million confirmed cases and 6.9 million deaths reported globally [5].

Scientific articles that began to appear almost from the first day of the pandemic described the symptoms accompanying the disease, including the very characteristic loss of smell and taste [6,7,8]. Subsequently, the effects of SARS-CoV-2 virus infection on the respiratory [9], circulatory [10], and nervous [11] systems were reported. More recently, scientific reports have also begun to emerge on the changes in the monthly cycle that have been observed in women with COVID-19; however, the research on this topic is still too scarce to draw definitive conclusions [12,13,14,15,16].

Emerging scientific evidence suggests that SARS-CoV-2 infection, the COVID-19 vaccination, and/or the psychological stress of the pandemic may affect a woman’s monthly cycle [17,18,19,20,21]. It is also already known that some viral infections correlate with changes in the female reproductive system, such as the duration of the menstrual cycle or the volume of menstruation [20]. A study by Phelan et al. [21] and Demir et al. [17] showed that the degree of stress and anxiety caused by the COVID-19 pandemic was sufficiently high to affect some of the menstrual cycle characteristics in women. The COVID-19 pandemic undoubtedly had an adverse impact on almost every aspect of human life, including the mental and reproductive health of the human population [22,23,24]. Therefore, the WHO [25] recommended that sexual and reproductive health services, including family planning, were essential services that should continue during the SARS-CoV-2 pandemic. 

A woman’s monthly cycle is a sign of health, fertility, and reproductive capacity. The menstrual cycle in women is characterised by high variability in cycle length (26–35 days), 5-day menses, a fertile phase from 5 days before to the day of ovulation, and low fertility which is dependent on cycle length and age [26]. The basis of a well-functioning monthly cycle is the highly coordinated hypothalamic–pituitary–ovarian (HPO) axis in combination with complex hormonal interactions as well as inflammatory and immunological mediators [27,28]. Psychological stress can activate the hypothalamic–pituitary–adrenal axis (HPA), inducing an inhibitory effect on the hypothalamic–pituitary–gonadal axis (HPG), which is connected with reduced pulsation of the gonadotropin-releasing hormone (GnRH). This reduced GnRH secretion from the hypothalamus inhibits the release of the follicle-stimulating hormone (FSH) and luteinising hormone (LH) as well as impeding follicular development. This has the effect of inhibiting ovarian secretion of oestrogen and progesterone [12,29,30,31] and can result in functional hypothalamic amenorrhoea (FHA) and a chronic lack of ovulation that is not due to an underlying organic cause [20,32,33,34,35,36,37]. Acute viral infections, including SARS-CoV-2, may also be associated with a disruption to the monthly cycle due to their effects on the hypothalamic–pituitary–ovarian–endometrial axis and mechanisms including immune dysregulation and direct inflammation of the ovaries [27,28,29].

Due to the fact that there is still little known about the effects of the SARS-CoV-2 virus on the female reproductive system, the authors of this study decided to add more information concerning the topic, obtained from women who started post-COVID-19 physiotherapy treatment, and in this way the authors have tried to fill the gaps about the impact of COVID-19 on the menstrual cycle in women. Therefore, the aim of this study was to assess the prevalence of changes in the women’s monthly cycles related to COVID-19, as well as to analyse correlations between dependent variables relating to changes in the monthly cycle and independent variables relating to other factors, such as age, weight, number and type of vaccinations, and time since illness. Additionally, this study assesses correlations between dependent variables relating to the monthly cycle and COVID-19 symptoms occurring during the course of the illness and persisting after the illness (long COVID).

## 2. Materials and Methods

### 2.1. Participants

This study involved 113 women who had suffered from COVID-19. The mean age of the subjects was 36.34 years ± standard deviation 9.10 years. The average height of the women was 167 ± 3.86 cm, body weight was 69.87 ± 6.86 kg, and body mass index (BMI) was 24.81 ± 2.16 kg/m^2^. Most of the women (71.68%) had been vaccinated against COVID-19. From the total sample, 53.10% were vaccinated with three doses. Two doses had been given to 12.39% of the subjects and only one dose to 6.19% of the women. Only 5.31% of the women had contracted COVD-19 before receiving the vaccination, while 66.37% had contracted the disease while already vaccinated. The majority had received the Pfizer vaccine (64.60%), while there were four respondents each for the Astra and Johnson vaccines, representing 3.54% each.

The inclusion criteria for the study were a history of COVID-19 and the presence of a monthly cycle. Women who became pregnant or gave birth during the pandemic were excluded from the study, as were women who were not menstruating for any reason, including using contraceptives, being menopausal, or having a medical condition that could have affected their monthly cycles. The authors of this research only had access to women who came to a clinic at the start of their post-COVID-19 physiotherapy treatment.

### 2.2. Study Design

This study was conducted as interviews with each woman during visits to a clinic prior to the start of their post-COVID-19 physiotherapy treatment. The reasons for patients presenting for this type of physiotherapy vary greatly. Those who qualified for the study in the publication were suffering mainly from lower tolerance to exercise, shortness of breath, excessive fatigue during physical activities, and even weakness and fatigue during routine daily activities, none of which they had experienced before their illness. Other reported problems included musculoskeletal pains and headaches. Undertaking physiotherapy was also seen as a prophylactic measure for the patients following the COVID-19 illness. Before starting therapy, an interview was always conducted as part of the qualifying visit for rehabilitation (indications and contraindications) and additional questions were asked, including those regarding health problems following COVID-19. As the literature on COVID-19 disease survivorship draws attention to the possible impact of this disease on the female reproductive system, in addition to routine questions during the interview, the authors of this manuscript also asked about the characteristics of the monthly cycle and any changes in the monthly cycle that the women noticed after their COVID-19 illness. The study period was from March 2022 to December 2022.

### 2.3. Statistical Analyses

Statistical analysis was performed using Spearman rho correlations. The next analysis looked for predictors of disorders in the monthly cycle by means of multiple regression analyses. There were eight disorders under study; however, to avoid performing eight regression analyses separately for each of these disorders, a joint aggregate index of the severity of the disorders was computed. This general index of the disorders in the monthly cycle was used as the dependent variable in two sets of multiple regression analyses. In both sets, main explaining predictors were analysed; they were entered by means of multiple regression. Results of both sets of regression analyses were compared as sensitivity analysis.

## 3. Results

**Figure 1:**
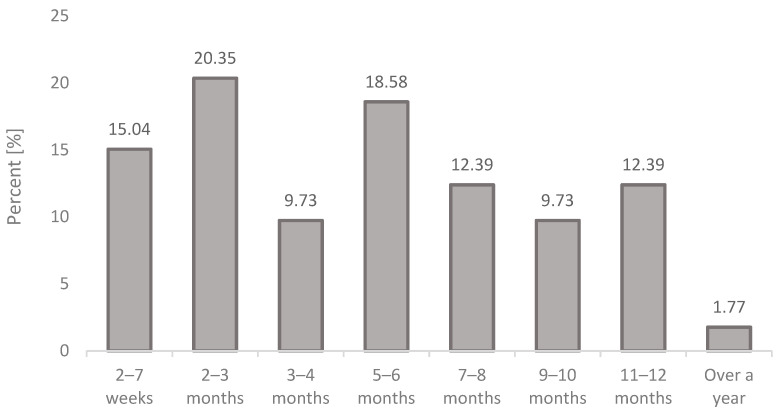
Time period from the onset of COVID-19.

Among the group of women, the time period from the onset of COVID-19 to the time of this research, i.e., the collection of information on possible changes in the monthly cycle related to the COVID-19 disease noted by the respondents, varied. The elapsed time between the onset of COVID-19 and the date of the interview about changes in the women’s monthly cycles is presented in Figure 1.

Almost all the patients had been treated at home, with only a small proportion of women (2.65%) being treated in hospital. The vast majority of women (81.42%) did not report any changes due to COVID-19 in their monthly cycles, while 18.58% of them observed changes in their cycles. The study showed that the more COVID-19 symptoms a woman had, the more often she noted changes in her monthly cycle, *p* < 0.001. The same relationship was also found for persistent long COVID symptoms, *p* < 0.001. The types of monthly cycle changes noted by women are summarised in Table 1.

Correlation analysis showed that the longer the time lapse since the COVID-19 infection, the less frequently changes in the monthly cycle were recorded (*p* < 0.01) (Table 2). It was shown that when a woman had been treated in hospital, because the course of the disease had been more severe, changes in the monthly cycle were recorded more frequently (*p* < 0.01) compared with the cases of the women who had been treated at home. Other changes in the monthly cycle observed and reported by the respondents correlated with the various factors presented in Table 2. These were mainly more severe headaches correlating positively with the woman’s age, indicating that the older the respondent was, the more often she reported these headaches during monthly bleeding in connection with COVID-19 history (*p* < 0.01) and the less often she had a regular period (*p* < 0.01) compared with the usual pattern. Correlation analysis also showed that obesity, like age, was positively associated with more severe headaches (*p* < 0.01) and negatively associated with monthly cycle regularity (*p* < 0.01) (Table 2).

In relation to vaccinations, it was shown that the more vaccinations a woman had received, the less frequently she experienced changes in her monthly cycle (*p* < 0.05). Reports of severe headaches (*p* < 0.05) and increased pain in the abdomen and lower abdomen (*p* < 0.05) were less frequent, as was an extension to the number of days menstrual bleeding lasted compared with the norm for the particular woman. When comparing the two groups of women, i.e., vaccinated and unvaccinated against COVID-19, it was observed that unvaccinated women were more likely to report a prolonged number of days of bleeding (*p* < 0.05). However, in relation to the type of vaccine received, it was found that those vaccinated with the Pfizer/BioNTech preparation were less likely (*p* < 0.01) to report changes in the length of monthly bleeding compared with women vaccinated with the AstraZeneca or Johnson & Johnson preparations.

When COVID-19 symptoms that were reported by women as occurring during and persisting after the illness (long COVID) were correlated with changes in the monthly cycle overall, statistical significance and a positive correlation were noted for dizziness (*p* < 0.05), sleep disturbance (*p* < 0.01), abdominal pain (*p* < 0.01), and fatigue and weakness (*p* < 0.01) (Table 3). More severe headaches during menstruation correlated positively with post-illness dizziness (*p* < 0.01) and perceived fatigue and weakness (*p* < 0.01). The change in the number of bleeding days and its prolongation, which positively correlated with post-disease persistent dizziness (*p* < 0.05) and general fatigue and weakness (*p* < 0.05), is also noteworthy. Sleep disturbance (*p* < 0.05) and persistent joint and muscle pain were also positively correlated with a disruption to monthly cycle regularity (Table 3).

The next analysis looked for predictors of the disorders in the monthly cycle by means of multiple regression analyses. There were eight disorders under study: increased pain in the abdomen and lower abdomen during menstruation; more severe headaches during menstruation; tearfulness/increased depression during menstruation; increased joint and muscle pain during menstruation; increased spotting during the cycle, followed by increased menstrual bleeding; heavy bleeding during menstruation; changes in the number of days of bleeding—prolonged; and disruption to cycle regularity compared with the woman’s own normal pattern. To avoid performing eight regression analyses separately for each of these disorders, a joint aggregate index of the severity of the disorders was computed. The aggregation was performed by means of a factor analysis extracting the first unrotated factor [37]. This had an eigenvalue of 2.90 and explained 28.61% of the variance in the symptoms. This factor was used as the general index of the disorders.

This general index of the disorders in the monthly cycle was used as the dependent variable in two sets of multiple regression analyses. In both sets, the following main explaining predictors were analysed: headaches, dizziness, sleep disturbance, loss of appetite, stomach-ache, fatigue and weakness, and joint and muscle pain. They were entered by means of multiple regression. In the first set, control variables were entered first: age [years], body weight [kg], obesity, height [cm], no. of vaccinations, COVID-19 after/before vaccinations, and time since onset of COVID-19. The main predictors were entered afterwards. In the second analysis, only the main predictors without the control variables were entered.

The results of both sets of regression analyses were compared as a sensitivity analysis. Sensitivity analysis consists in performing analysis of some variants that differ in important respects [38]. In this analysis, the authors compared the impact of the independent variables on disorders in the monthly cycle with and without the control variables mentioned above. Three predictors remained significant in both analyses: stomach-ache (*p* < 0.001), fatigue and weakness (*p* < 0.001), and joint and muscle pain (*p* < 0.05). One (loss of appetite) was only important (*p* < 0.01) in the analysis with control variables. As for the control variables themselves, obesity was a predictor of disorders in the monthly cycle (*p* < 0.01) (Table 4).

## 4. Discussion

The effect of COVID-19 on a woman’s menstrual cycle is largely unknown; however, severe illness caused by a viral infection, including SARS-CoV-2, can cause hypothalamic hypogonadism leading to an absence of menstruation or infrequent menstrual bleeding [22,23,39].

The results of our research indicated that most of the women surveyed had no changes in their monthly cycles; however, it should be borne in mind that these were mostly young women who had relatively mild cases of the disease and had been treated at home. The results of this research also showed that the longer the time lapse since COVID-19 infection, the less frequently the women reported changes in their monthly cycles. A variety of factors were also found to influence changes in the women’s menstrual cycles after COVID-19, most notably age and obesity. Both factors correlated positively with both severe headaches during menstruation and disruption to the regularity of the monthly cycle in relation to what the woman would consider as normal. The changes in the monthly cycles observed by the women were mostly related to increased pain in the abdomen and lower abdomen, headaches, and increased pain in the joints and muscles. Approximately 10 percent of the women reported that their monthly cycles were longer and that there was a disruption in regularity from the pattern considered normal for the woman in question. In the statistical analysis, the impact of the independent variables on disorders in the monthly cycle with and without the control variables was additionally compared. The following three predictors remained significant: stomach-ache, fatigue and weakness, and joint and muscle pain. One (loss of appetite) was only important in the analysis with control variables. As for the control variables themselves, obesity was a predictor of disorders in the monthly cycle. 

Vaccination seems to have a kind of ‘protective character’ on the occurrence of changes in the menstrual cycle, as our study showed that the more vaccinations a woman received, the less frequently she reported changes in her monthly cycle. In addition, unvaccinated women were more likely to report an increase in the number of days of bleeding. In our study, the number of women treated in hospital was small, approximately 3%, and therefore no definitive comparisons can be made between women treated at home and those treated in hospital. However, the women in our study who suffered more severe cases of the disease and were therefore treated in hospital reported more problems with their menstrual cycles.

Our study also analysed the monthly cycle and its correlation with the persistence of symptoms of so-called long COVID. Scientific studies are providing increasing evidence on the symptoms and complications that persist for several weeks or more after the SARS-CoV-2 infection, the so-called long COVID. These are mainly pulmonary, cardiovascular, haematological, neuropsychiatric, dermatological, gastrointestinal, or skeletal complications, which all carry health risks for COVID-19 survivors [2]. Although current knowledge concerning long-term COVID-19 symptoms, complications, and complaints is constantly increasing, there is still a need to find answers to many questions related to this issue. Our own research has shown a significant correlation between the sum of all persistent long COVID symptoms and changes in the monthly cycle. The women indicated that disturbances in the monthly cycle correlate positively with such persistent long COVID symptoms as sleep disturbances, dizziness, fatigue and weakness, and abdominal pain. Of note, prolongation of menstrual bleeding positively correlates with women’s perceived fatigue and weakness following the illness.

In both this research and other scientific literature [39], it was observed that women who reported changes to their monthly cycles after SARS-CoV-2 infection also reported more COVID-19 symptoms. In a review of the literature [34] aimed at analysing the results on the correlation between the SARS-CoV-2 infection and menstrual cycle changes, it was found that reports from several studies indicated changes in the volume of monthly bleeding and changes in cycle length as consequences of the SARS-CoV-2 infection. In the research by Khan et al. [18], it was shown that women who reported changes in their monthly cycles (most commonly irregular menstruation as well as infrequent menstruation) also reported more symptoms of COVID-19 and were more likely to be overweight or obese. Phelan et al. [21] reported changes in women’s monthly cycles, for example, additional menstrual bleeding, more painful menstruation compared with before the pandemic, and missed periods that had not previously occurred. In the research by Muharan et al. [40], there was an increase in the number of patients with cycles of >32 or <24 days and a significant increase in menstrual irregularity and heavy menstrual bleeding. Li et al. [12] found that almost one fifth of regularly menstruating women who were hospitalised with the SARS-CoV-2 infection had changes in their monthly cycles, mainly manifesting as a significant decrease in the volume of blood lost during menstruation or a prolongation of the monthly cycle. The above findings were questioned in the research of Danesh et al. [41], in which the authors found that only about 6–7% of people infected with SARS-CoV-2 require hospitalisation and hospitalised COVID-19 patients are more likely to have multimorbidity, obesity, or metabolic syndrome that may cause menstrual disorders. Madann et al. [42] also found that menstrual changes were observed more frequently in COVID-19 patients with systemic complications due to the illness. Ding et al. [43] stated that the menstrual cycle, menstrual volume, menstrual cycle phase, and history of painful menstruation were similar with no significant differences between women with mild and severe COVID-19. 

The effect of a COVID-19 vaccination on a woman’s monthly cycle has also become a topic of discussion and of many studies [44]. Grandone et al. [45], based on a review of the available literature focusing on adverse events in recipients of vaccines against SARS-CoV-2, reported more side effects in women than in male recipients of certain anti-COVID-19 vaccines. For example, thrombotic events, both arterial and venous, were mostly observed in young women receiving viral-vector vaccines, as were haemorrhagic complications. Women have a significantly higher risk of bleeding episodes lasting 3–4 weeks: 37% reported prolonged duration of skin bleeds, 14% nose bleeds, and 26.5% gingival bleeds, as well as abnormal menstrual cycle (delayed/increased haemorrhages or pain) in 0.98% of mRNA and 0.68% of viral-vector vaccine recipients. An increase in usual cycle length has been observed following vaccination with both the mRNA and COVID-19 adenovirus vector-based vaccines. There are a few probable mechanisms to explain this observed link, including immune effects on sex hormones and systemic inflammatory responses that may trigger further reactions in target organs [46]. The immune response induced by both mRNA-containing and adenovirus vector-containing vaccines can transiently affect the HPO axis, which can lead to menstrual disorders [47]. The results obtained by Bouchard et al. [48] did not confirm any significant differences in the characteristics of the monthly cycle’s basic parameters, such as length of cycle, bleeding length, and luteal phase length, before and after a COVID-19 vaccination. Edelman et al. [13] found that vaccination against COVID-19 was associated with an increase in the length of the menstrual cycle of less than one day, but not with an increase in menstrual bleeding. Most women who participated in the studies by Al.-Najjar et al. [15] and Rodríguez Quejada [39] reported that COVID-19 had caused changes in the amount of blood loss during menstruation, and changes in the number of days of bleeding as well as the number of days of a monthly cycle. Research by Laganà et al. [49] reported that the most common changes were shorter or longer monthly cycles and heavier monthly bleeding. Studies by Dabbousi et al. [50] also found that the number of women having regular monthly cycles decreased after they received the vaccine. In research by Baena-Garcia et al. [51], it was found that COVID-19 vaccines with the adenovirus vector appear to be linked more with menstrual cycle changes than mRNA vaccines. In a study by Muhaidat et al. [52], more than half of the women reported changes in their monthly cycles after vaccination, of which 46.7% experienced these after the first dose. In research by Gibson et al. [53], an association was found between the vaccine dose and mean cycle length, the variation in which depended on the phase in which the dose was given. Vaccination in the follicular phase with the mRNA vaccine was associated with an increase in mean cycle length in the next few cycles. 

The COVID-19 pandemic affected women’s reproductive health, and further research focused on this topic is needed to compare current and future research findings and better elucidate the potential physiological mechanisms behind the changes in a woman’s monthly cycle that may occur after SARS-CoV-2 virus infection. 

### Limitations

The number of subjects reporting changes in their menstrual cycles was limited to 21 women. The authors of this manuscript are aware that it is difficult to perform a significant analysis on this number of subjects; however, we believe that our findings show some interesting information that is worthy of further research on larger groups of women. The study conducted was retrospective, which may have contributed to some errors in the assessments of the monthly cycles declared by the women surveyed. In addition, during the pandemic, the monthly cycle, at any one time, was influenced by a number of factors, including viral infection, stress, and/or vaccination, so it is difficult to assess conclusively whether it was the virus or other factors affecting the woman’s body that caused changes in the menstrual cycle. There is a further need for research on the impact of COVID-19 on the menstrual cycles of women to fill the gaps in the knowledge. The authors of further research should take into account various factors, including, for example, the association of demographic and lifestyle factors, for example smoking status, psychological stress in the workplace, caffeine consumption, etc.

## 5. Conclusions

There were no changes in the monthly cycle among most of the women examined who have had COVID-19. Women who declared that COVID-19 affected their monthly cycles most often indicated increases in abdominal, lower abdominal, and joint and muscle pain, as well as in the severity of headaches during monthly bleeding. A small percentage of women indicated that their monthly cycles were longer and their regularity disrupted. Women who reported more symptoms of COVID-19 were more likely to report changes in their menstrual cycles occurring after SARS-CoV-2 infection compared with women whose disease course was mild. Considering the impact of the independent variables on disorders in the monthly cycle with and without the control variables, these three predictors remained significant: stomach-ache, fatigue and weakness, and joint and muscle pain. One (loss of appetite) was only important in the analysis with control variables. As for the control variables themselves, obesity was a predictor of disorders in the monthly cycle. 

The more vaccinations a woman received, the less frequently changes in the monthly cycle were reported. Unvaccinated women were more likely to report an increase in the number of days of monthly bleeding as an impact of COVID-19, compared with vaccinated women.

## Figures and Tables

**Table 1:** Changes in the monthly cycle related to COVID-19 recorded by the women.

	N = 113	Percent [%]
Number of women who reported changes in their monthly cycles:	21	18.58
Details of the type and number of changes in the monthly cycle reported by the surveyed women:	The women reported 44 changes (in total)	
➢ Increased pain in abdomen and lower abdomen	17	38.64
➢ More severe headaches during menstruation	7	15.91
➢ Increased joint and muscle pain during menstruation	6	13.64
➢ Change in number of days of bleeding—prolonged	4	9.09
➢ Disruption to cycle regularity compared with the woman’s own normal pattern	4	9.09
➢ Increased spotting during the cycle, followed by increased monthly bleeding	3	6.82
➢ Heavy bleeding during menstruation	2	4.55
➢ Tearfulness/increased depression during menstruation	1	2.27
Number of women who reported no changes to their monthly cycles:	92	81.42

**Table 2:** Analyses of correlations between dependent variables relating to changes in the monthly cycle and independent variables relating to other factors, such as age, weight, number and type of vaccinations, and time since illness. Spearman rho correlations.

	Age [Years]	Body Weight [kg]	Obesity	Height [cm]	No. of Vaccinations	Vaccinated/Unvaccinated	Type of Vaccination	COVID-19 Occurred after Vaccination/before Vaccination	Time since the Onset of COVID-19	Place of Treatment—Home or Hospital
Occurrence of any changes in the monthly cycle related to COVID-19	0.13	0.01	0.11	−0.05	−0.21 *	−0.20 *	0.01	0.11	−0.35 **	0.35 **
Increased pain in the abdomen and lower abdomen	0.08	0.04	0.13	−0.06	−0.20 *	−0.18	−0.01	0.10	−0.26 **	0.39 **
More severe headaches during menstruation	0.24 *	0.17	0.24 **	−0.01	−0.21 *	−0.16	0.06	0.06	−0.18	0.19 *
Tearfulness/increased depression during menstruation	0.09	0.12	−0.01	0.11	−0.13	−0.15	.	.	−0.08	−0.02
Increased joint and muscle pain during menstruation	0.09	−0.04	−0.03	−0.09	−0.06	−0.11	0.06	0.06	−0.22 *	0.45 **
Increased spotting during the cycle followed by increased monthly bleeding	0.03	−0.06	−0.02	−0.04	0.02	−0.02	0.05	0.05	−0.15	0.03
Heavy bleeding during menstruation	−0.05	−0.16	−0.02	−0.07	−0.03	−0.06	0.04	0.03	−0.16	0.40 **
Change in number of days of bleeding—prolonged	−0.03	−0.06	−0.03	−0.04	−0.21 *	−0.20 *	−0.34 **	0.03	−0.07	−0.03
Disruption to cycle regularity compared with the woman’s own normal pattern	−0.27 **	−0.05	−0.25 **	0.10	0.09	0.08	0.03	−0.15	0.05	−0.04

* *p* < 0.05; ** *p* < 0.01.

**Table 3:** Analyses of correlations between dependent variables relating to the monthly cycle and COVID-19 symptoms occurring during the course of the illness and persisting after the illness (long COVID).

	Any Changes in the Monthly Cycle Associated with COVID-19	Increased Pain in Abdomen and Lower Abdomen during Menstruation	More Severe Headaches during Menstruation	Tearfulness/Increased Depression during Menstruation	Increased Joint and Muscle Pain during Menstruation	Increased Spotting during the Cycle, Followed by Increased Menstrual Bleeding	Heavy Bleeding during Menstruation	Change in Number of Days of Bleeding—Prolonged	Disruption to Cycle Regularity Compared with the Woman’s Own Normal Pattern
Disorders experienced after COVID-19 (long COVID) and changes in the monthly cycle									
Headaches	−0.08	−0.07	−0.04	−0.02	−0.04	−0.03	−0.02	−0.03	−0.03
Dizziness	0.23 *	0.27 **	0.30 **	−0.02	0.14	−0.04	−0.03	0.19 *	−0.04
Sleep disturbance	0.40 **	0.31 **	0.02	−0.03	0.16	0.29 **	0.16	0.08	0.23 *
Loss of appetite	0.06	0.08	−0.04	−0.02	0.21 *	−0.03	0.40 **	−0.03	−0.03
Stomach-ache	0.29 **	0.34 **	0.10	−0.02	0.30 **	0.21 *	−0.03	0.17	−0.05
Fatigue and weakness	0.52 **	0.46 **	0.42 **	0.15	0.30 **	0.15	0.22 *	0.20 *	0.10
Joint and muscle pain	0.01	−0.09	−0.06	−0.02	−0.05	−0.04	−0.03	−0.04	0.19 *

* *p* < 0.05; ** *p* < 0.01.

**Table 4:** Results of multiple regression analyses with and without control variables.

Model	Predictors	B	Beta	*t*	*p*
With control variables: *R^2^* = 0.55 *p* < 0.001	Age [years]	0.00	−0.03	−0.31	0.758
	Body weight [kg]	0.02	0.14	1.19	0.238
	Obesity	2.22	0.43	2.47	**0.016**
	Height [cm]	−0.01	−0.05	−0.41	0.681
	No. of vaccinations	−0.01	0.00	−0.04	0.968
	COVID-19 after/before vaccinations	0.03	0.01	0.11	0.909
	Time since onset of COVID-19	−0.01	−0.03	−0.25	0.803
	Headaches	0.55	0.07	0.57	0.567
	Dizziness	−0.83	−0.19	−1.18	0.243
	Sleep disturbance	−0.35	−0.12	−1.07	0.291
	Loss of appetite	1.26	0.24	2.85	**0.006**
	Stomach-ache	1.97	0.46	4.79	**<0.001**
	Fatigue and weakness	0.97	0.41	3.67	**<0.001**
	Joint and muscle pain	−2.62	−0.36	−2.36	**0.021**
Without control variables: *R^2^* = 0.35 *p* < 0.001	Headaches	−1.11	−0.18	−2.20	0.030
	Dizziness	0.85	0.18	2.12	0.037
	Sleep disturbance	0.16	0.05	0.60	0.552
	Loss of appetite	0.02	0.00	0.05	0.962
	Stomach-ache	1.24	0.28	3.39	**0.001**
	Fatigue and weakness	0.98	0.44	5.20	**<0.001**
	Joint and muscle pain	−0.76	−0.16	−1.98	**0.050**

*R*^2^: coefficient of multiple determination (tells what proportion of the dependent variable is explained by all predictors); *p*: probability level for the model (tells whether the model is statistically significant); B: unstandardised regression coefficients; Beta: standardised regression coefficients; t: *t*-statistic for the predictor; *p*: probability (significance) for the predictor.

## Data Availability

The datasets used and/or analysed during the current study are available from the corresponding author on reasonable request.
